# Prevalence of Disability and Use of Accommodation Among US Allopathic Medical School Students Before and During the COVID-19 Pandemic

**DOI:** 10.1001/jamanetworkopen.2023.18310

**Published:** 2023-06-14

**Authors:** Karina Pereira-Lima, Melissa A. Plegue, Ben Case, Bonnielin K. Swenor, Kurt Herzer, Rylee Betchkal, Lisa M. Meeks

**Affiliations:** 1 Department of Neurology, University of Michigan Medical School, Ann Arbor; 2 Susan B. Meister Child Health Evaluation and Research Center, Department of General Pediatrics, University of Michigan Medical School, Ann Arbor; 3 Meeks Research Lab, University of Michigan Medical School, Ann Arbor; 4 Johns Hopkins Disability Health Research Center, Baltimore, Maryland; 5 Johns Hopkins School of Medicine, Baltimore, Maryland; 6 Department of Learning Health Sciences, University of Michigan Medical School, Ann Arbor; 7 Center for Disability Health and Wellness, University of Michigan Medical School, Ann Arbor

## Abstract

This survey study assesses self-disclosures of disability, disability types, and accommodation needs reported by US allopathic medical schools in 2021 vs 2015 and 2019.

## Introduction

Data from the first 2 waves of a longitudinal prevalence study of institutionally reported disability in US allopathic programs indicated growth of medical students with disabilities (SWDs).^1,2^ Access to needed accommodations is critical to medical trainees with disabilities,^3^ and changes catalyzed by the COVID-19 pandemic and remote delivery of curriculum^4^ carry potential implications for SWDs’ accommodation needs. We examined changes in disability disclosure and accommodation use longitudinally and during the pandemic.

## Methods

Between July 2021 and January 2022, in accordance with previous methods,^1,2^ a web-based survey (eMethods 1 in Supplement 1) was sent to institutionally designated disability resource professionals at 154 fully accredited US allopathic medical schools. The University of Colorado School of Medicine Institutional Review Board deemed this study exempt from review and waived the informed consent requirement because aggregated deidentified data were used. We followed the AAPOR reporting guideline.

Differences in national proportions of SWDs and disability types, calculated by combining data from all schools that provided complete information (eMethods 2 in Supplement 1), were compared using identical survey items across the 2015,^1^ 2019,^2^ and 2021 waves. Pairwise comparisons between the proportions of disability and accommodation use across all waves were performed using *z* tests, with 2-sided *P* < .05 indicating statistical significance. *P* values were adjusted for multiple comparisons using Bonferroni correction. Sensitivity analysis was performed using data from schools represented in all waves. Analyses were conducted in R 3.5.1 (R Core Team).

## Results

Fifty-six schools responded to the 2021 survey, identifying 2125 SWDs of 36 322 students (5.9%). This number indicated continued and significant increase in SWDs over time (2015: 1596 [2.8%]; 2019: 2367 [4.6%]; *P* < .001) (Table 1).

**Table 1.** Proportion of Allopathic Medical Students Disclosing Disabilities From 2015 to 2021

	Medical school sample, No. (%) [95% CI]^a^			Adjusted *P* value^b^		
	2015 (n = 90 schools; n = 57 794 students)^c^	2019 (n = 82 schools; n = 51 263 students)^c^	2021 (n = 56 schools; n = 36 322 students)^c^	2015 vs 2019	2015 vs 2021	2019 vs 2021
School-reported prevalence of disability types disclosed by full student body						
Overall^d^	1596 (2.8) [2.6-2.9]	2367 (4.6) [4.4-4.8]	2125 (5.9) [5.6-6.1]	<.001	<.001	<.001
ADHD	522 (0.9) [0.8-1.0]	697 (1.4) [1.3-1.5]	629 (1.7) [1.6-1.9]	<.001	<.001	<.001
Learning disabilities	358 (0.6) [0.6-0.7]	430 (0.8) [0.8-0.9]	277 (0.8) [0.7-0.9]	<.001	.24	>.99
Psychological disabilities^e^	322 (0.6) [0.5-0.6]	734 (1.4) [1.3-1.5]	701 (1.9) [1.8-2.1]	<.001	<.001	<.001
Deaf or hard of hearing	35 (0.1) [0.0-0.1]	29 (0.1) [0.0-0.1]	39 (0.1) [0.1-0.2]	>.99	.35	.21
Visual disabilities	46 (0.1) [0.1-0.1]	54 (0.1) [0.1-0.1]	37 (0.1) [0.1-0.1]	>.99	>.99	>.99
Mobility disabilities	42 (0.1) [0.1-0.1]	84 (0.2) [0.1-0.2]	69 (0.2) [0.2-0.2]	<.001	<.001	>.99
Chronic health disabilities	209 (0.4) [0.3-0.4]	437 (0.9) [0.8-0.9]	386 (1.1) [1.0-1.2]	<.001	<.001	.04
Other functional impairment^f^	62 (0.1) [0.1-0.1]	69 (0.1) [0.1-0.2]	52 (0.1) [0.1-0.2]	>.99	>.99	>.99
School-reported proportion of disability types disclosed by SWDs						
ADHD	522 (32.7) [30.4-35.1]	697 (29.4) [27.6-31.3]	629 (29.6) [27.7-31.6]	.70	>.99	>.99
Learning disabilities	358 (22.4) [20.4-24.6]	430 (18.2) [16.6-19.8]	277 (13.0) [11.6-14.5]	.02	<.001	<.001
Psychological disabilities^e^	322 (20.2) [18.2-22.2]	734 (31.0) [29.1-32.9]	701 (33.0) [31.0-35.0]	<.001	<.001	>.99
Deaf or hard of hearing	35 (2.2) [1.5-3.0]	29 (1.2) [0.8-1.8]	39 (1.8) [1.3-2.5]	.43	>.99	>.99
Visual disabilities	46 (2.9) [2.1-3.8]	54 (2.3) [1.7-3.0]	37 (1.7) [1.2-2.4]	>.99	.48	>.99
Mobility disabilities	42 (2.6) [1.9-3.5]	84 (3.5) [2.8-4.4]	69 (3.2) [2.5-4.1]	>.99	>.99	>.99
Chronic health disabilities	209 (13.1) [11.5-14.8]	437 (18.5) [6.9-20.1]	386 (18.2) [16.5-19.9]	<.001	<.001	>.99
Other functional impairment^f^	62 (3.9) [3.0-5.0]	69 (2.9) [2.3-3.7]	52 (2.4) [1.8-3.2]	>.99	.28	>.99

Abbreviations: ADHD, attention-deficit/hyperactivity disorder; SWD, student with disability.

^a^ Percentages may add up to more than 100% due to some schools reporting students with multiple types of disabilities.

^b^ *P* values of pairwise comparisons using *z* tests. *P* values were adjusted for each set of comparisons using Bonferroni correction.

^c^ Included only schools that provided complete data on both prevalence of disability types disclosed by all students and proportion of disability types disclosed by SWDs. Regions for schools included in each study wave were as follows: 2015 wave: Central (21 [23.3%]), Northeast (26 [28.9%]), Southern (29 [32.2%]), Western (14 [15.6%]); 2019 wave: Central (25 [30.5%]), Northeast (24 [29.3%]), Southern (19 [23.2%]), Western (14 [17.1%]). 2021 wave: Central (17 [30.4%]), Northeast (16 [28.6%]), Southern (11 [19.6%]), Western (12 [21.4%]).

^d^ Results from sensitivity analyses of prevalence estimates for the 38 schools that responded in all 3 waves were as follows: 2015: 2.94 (95% CI, 2.73-3.16), 2019: 5.01 (95% CI, 4.75-5.29), and 2021: 6.22 (95% CI, 5.93-6.52).

^e^ Psychological disabilities included adjustment disorder, anxiety disorder, obsessive-compulsive disorder, posttraumatic stress disorder, bipolar disorder, depression, eating disorder, cognitive disorder, schizophrenia or other psychotic disorder, and other psychological disabilities.

^f^ Other functional impairment included missing limbs, traumatic brain injury, and stuttering.

Overall, the population of students reporting attention-deficit/hyperactivity disorder (ADHD; 2015: 522 [0.9%]; 2019: 697 [1.4%]; 2021: 629 [1.7%]), chronic health disabilities (2015: 209 [0.4%]; 2019: 437 [0.9%]; 2021: 386 [1.1%]), and psychological disabilities (2015: 322 [0.6%]; 2019: 734 [1.4%] [2019]; 2021: 701 [1.9%]) significantly increased across 3 waves (*P* = .04 to <.001). Within the population of SWDs, only the proportion of those who disclosed learning disabilities (18.2% vs 13.0%; *P* < .001) significantly declined from 2019 to 2021 (Table 1).

The percentage of SWDs requesting most types of didactic testing–related accommodations significantly decreased from 2019 to 2021 (reduced distraction: 57.9% vs 51.1%, *P* < .001; private environment: 8.7% vs 6.2%, *P* = .02; additional examination time: 74.9% vs 66.9%, *P* < .001). For nontesting didactic accommodations, significant increases from 2019 to 2021 were observed for recorded lectures (5.6% vs 10.2%; *P* < .001), whereas requests for housing (4.6% vs 2.2%; *P* < .001) significantly decreased (2.6% vs 1.6%; *P* = .01). Clinical-testing accommodations significantly increased from 2019 to 2021 (extra time: 37.8% vs 45.1%, *P* < .001; reduced distraction: 31.0% vs 42.4%, *P* < .001). For clinical accommodations, significant increases from 2019 to 2021 were observed only for release from clinical appointments (5.3% vs 7.7%, *P* = .02) (Table 2).

**Table 2.** Proportion of Allopathic Medical SWDs Using Accommodations From 2015 to 2021

	Medical school sample, No. (%) [95% CI]			Adjusted *P* value^a^		
	2015 (n = 89 schools; n = 1547 SWD)^b^	2019 (n = 80 schools; n = 2235 SWD)^b^	2021 (n = 56 schools; n = 2125 SWD)^b^	2015 vs 2019	2015 vs 2021	2019 vs 2021
Prevalence of SWDs requesting different types of accommodations						
Didactic (preclinical) testing accommodations						
Reduced-distraction environment	814 (52.6) [50.1-55.1]	1293 (57.9) [55.8-59.9]	1085 (51.1) [48.9-53.2]	.02	>.99	<.001
Private environment	197 (12.7) [11.1-14.5]	195 (8.7) [7.6-9.9]	132 (6.2) [5.2-7.3]	.001	<.001	.02
Additional examination time	1120 (72.4) [70.1-74.6]	1673 (74.9) [73.0-76.6]	1421 (66.9) [64.8-68.9]	>.99	.004	<.001
Examination format	NA^c^	51 (2.3) [1.7-2.9]	32 (1.5) [1.0-2.1]	NA^c^	NA^c^	.80
Breaks	98 (6.3) [5.2-7.7]	197 (8.8) [7.7-10.1]	185 (8.7) [7.5-10.0]	.07	.10	>.99
Didactic or preclinical nontesting accommodations						
Alternate format	42 (2.7) [2.0-3.7]	94 (4.2) [3.4-5.1]	91 (4.3) [3.5-5.2]	.43	.33	>.99
Assistance or service animal	13 (0.8) [0.4-1.4]	35 (1.6) [1.1-2.2]	44 (2.1) [1.5-2.8]	>.99	.08	>.99
Attendance	77 (5.0) [3.9-6.2]	135 (6.0) [5.1-7.1]	129 (6.1) [5.1-7.2]	>.99	>.99	>.99
Ergonomic evaluation or equipment	19 (1.2) [0.7-1.9]	54 (2.4) [1.8-3.1]	53 (2.5) [1.9-3.2]	.25	.18	>.99
Housing	6 (0.4) [0.1-0.8]	103 (4.6) [3.8-5.6]	47 (2.2) [1.6-2.9]	<.001	<.001	<.001
Livescribe smartpen	35 (2.3) [1.6-3.1]	56 (2.5) [1.9-3.2]	34 (1.6) [1.1-2.2]	>.99	>.99	>.99
Note taking	86 (5.6) [4.5-6.8]	117 (5.2) [4.3-6.2]	130 (6.1) [5.1-7.2]	>.99	>.99	>.99
Programmatic accommodations	NA^c^	59 (2.6) [2.0-3.4]	33 (1.6) [1.1-2.2]	NA^c^	NA^c^	.34
Recorded lectures	86 (5.6) [4.5-6.8]	125 (5.6) [4.6-6.6]	217 (10.2) [9.0-11.6]	>.99	<.001	<.001
Text to speech or speech to text	59 (3.8) [2.9-4.9]	49 (2.2) [1.6-2.9]	78 (3.7) [2.9-4.6]	.09	>.99	.10
Clinical testing accommodations						
Extra time on clinical-based examinations	NA^c^	844 (37.8) [35.7-39.8]	959 (45.1) [43.0-47.3]	NA^c^	NA^c^	<.001
Reduced-distraction environment	NA^c^	693 (31.0) [29.1-33.0]	900 (42.4) [40.2-44.5]	NA^c^	NA^c^	<.001
Clinical accommodations						
Simulation laboratory	NA^c^	7 (0.3) [0.1-0.6]	18 (0.8) [0.5-1.3]	NA^c^	NA^c^	.37
Assistive technology	NA^c^	37 (1.7) [1.2-2.3]	39 (1.8) [1.3-2.5]	NA^c^	NA^c^	>.99
CART or real-time captioning	27 (1.7) [1.2-2.5]	10 (0.4) [0.2-0.8]	21 (1.0) [0.6-1.5]	.001	.88	.65
Decelerated clinical year	13 (0.8) [0.4-1.4]	14 (0.6) [0.3-1.0]	17 (0.8) [0.5-1.3]	>.99	>.99	>.99
Intermediary or assistant for patient examination	NA^c^	6 (0.3) [0.1-0.6]	11 (0.5) [0.2-0.9]	NA^c^	NA^c^	>.99
Release from clinical appointments	NA^c^	118 (5.3) [4.4-6.3]	164 (7.7) [6.6-8.9]	NA^c^	NA^c^	.02
Release from overnight call	16 (1.0) [0.6-1.7]	49 (2.2) [1.6-2.9]	52 (2.4) [1.8-3.2]	.14	.03	>.99
Scribe application	9 (0.6) [0.3-1.1]	6 (0.3) [0.1-0.6]	1 (0.05) [0.01-0.3]	>.99	.04	>.99
Specialized clinical placement site	59 (3.8) [2.9-4.9]	85 (3.8) [3.0-4.7]	81 (3.8) [3.0-4.7]	>.99	>.99	>.99

Abbreviations: CART, Communication Access Real-Time Translation; NA, not applicable; SWD, student with disability.

^a^ *P* values of pairwise comparisons using *z* tests. *P* values were adjusted for each set of comparisons using Bonferroni correction.

^b^ Included only schools that provided complete data on the prevalence of SWDs, individual disability types, and accommodation use.

^c^ Referred to data that were not collected in the 2015 wave.

## Discussion

In 2021, allopathic medical schools reported a SWD prevalence of 5.9%, a 26.6% relative increase from 2019 and more-than-double growth since 2015, with significant increases in ADHD, chronic health disabilities, and psychological disabilities. The proportion of SWDs who reported learning disabilities significantly declined from 2019 to 2021. Despite the higher prevalence of SWDs, request for most didactic testing–related accommodations significantly decreased from 2019 to 2021. This decrease may be due to the remote delivery of curriculum during the pandemic, allowing students to create optimal learning and testing environments.^5^

Study limitations included potential underestimations due to nondisclosure of disability^6^ and a lower response rate. However, sensitivity analysis suggested similar prevalence estimates, and data were institutionally verified, increasing their accuracy.

Disability disclosure continues to increase among medical students, with unexpected decreases in reporting of learning disabilities and didactic-testing accommodations during the pandemic. Schools should consider enhancing accessible experiences for all learners (eg, through universal design approach). Future studies should evaluate whether changes in disability disclosure and accommodation requests remain with the return of in-person learning.

## Notes

Supplement 1. **eMethods 1.** Survey Questions Used in the Present Study **eMethods 2.** Number of Medical Schools Meeting Inclusion Criteria in Analyses for Outcomes Related to Disability Prevalence and Accommodation Use by Study Wave

Supplement 2.
Data Sharing Statement
