# Supplementary material for: Prevalence of Disability and Use of Accommodation Among US Allopathic Medical School Students Before and During the COVID-19 Pandemic
Source: JAMA Netw Open. 2023 Jun 14;6(6):e2318310. doi: 10.1001/jamanetworkopen.2023.18310 (PMC10267761; doi:10.1001/jamanetworkopen.2023.18310)
Supplement: Supplement 1. — eMethods 1. Survey Questions Used in the Present Study eMethods 2. Number of Medical Schools Meeting Inclusion Criteria in Analyses for Outcomes Related to Disability Prevalence and Accommodation Use by Study Wave [file jamanetwopen-e2318310-s001.pdf]

Supplemental Online Content

Pereira-Lima K, Plegue MA, Case B, et al. Prevalence of disability and use of accommodation among US allopathic medical school students before and during the COVID-19 pandemic. *JAMA Netw Open*. 2023;6(6):e2318310. doi:10.1001/jamanetworkopen.2023.18310

eMethods 1. Survey Questions Used in the Present Study

eMethods 2. Number of Medical Schools Meeting Inclusion Criteria in Analyses for Outcomes Related to Disability Prevalence and Accommodation Use by Study Wave

This supplemental material has been provided by the authors to give readers additional information about their work.

© 2023 Pereira-Lima K et al. *JAMA Network Open.*

eMethods 1. Survey questions used in the present study[a]

1. Please select the school for which you are submitting information

** Official LCME school names are provided.*

[Drop down list containing all accredited U.S. medical schools]

Name of person completing the form

[open-text response]

Email of person completing the form

[open-text response]

2. Please provide the total number of Medical Students that are currently registered with the Disability Services regardless of whether they currently receive accommodation

[open-text response]

3. Of the total medical students registered with disability services, how many are registered under each of the following disability categories? Please select only one category per student using the student's primary disability type. If the student's disability is not represented here, please write it in under the other option.

Acquired/Traumatic Brain Injury:	[open-text response]
Attention Deficit/Hyperactivity Disorder:	[open-text response]
Chronic health condition (e.g. lupus, arthritis, chronic back pain):	[open-text response]
Deaf and Hard of Hearing:	[open-text response]
Learning Disability:	[open-text response]
Mobility:	[open-text response]
Psychological Disability:	[open-text response]
Speech/Other Communication Disability:	[open-text response]
Vision - Low Vision:	[open-text response]
Other (please specify below)	[open-text response]
Please enter one line for each additional disability service and include the number of students in this category.	[open-text response]

4. Of all the medical students registered with disability services, how many were granted each of the following accommodations for the didactic portion of their education? Total number of responses may be greater than the

© 2023 Pereira-Lima K et al. *JAMA Network Open.*

total number of students registered with disability services, given that most students have more than one accommodation.

Assistance or Service Animal:	[open-text response]
Alternate Format (textbook conversion, other):	[open-text response]
Attendance:	[open-text response]
Environmental - Low Distraction:	[open-text response]
Environmental - Private:	[open-text response]
Ergonomic Evaluation or Equipment:	[open-text response]
Extra Exam Time:	[open-text response]
Exam Format Other (please enter number then describe below)	[open-text response]
Exam Format Other (please describe):	[open-text response]
Housing (Single Room, Release, Parking):	[open-text response]
Livescribe Pen:	[open-text response]
Notetaking:	[open-text response]
Programmatic Accommodation (e.g., decelerated curriculum) Please enter number then describe below:	[open-text response]
Programmatic Accommodation (please describe):	[open-text response]
Record Lectures:	[open-text response]
Test Breaks:	[open-text response]
Text to Speech/Speech to Text (Screenreader, Dragon):	[open-text response]

5. Of all the medical students registered with disability services, how many medical students were granted each of the following accommodations for the clinical portion of their education? Total number of responses may be greater than the total number of students registered with disability services, given that most students have more than one accommodation.

Ability to perform procedural skill or clinical competency in simulation lab:	[open-text response]
Assistive Technology (e.g., Specialized stethoscope otoscope, other devices):	[open-text response]
CART/Real-Time Captioning:	[open-text response]

© 2023 Pereira-Lima K et al. *JAMA Network Open.*

Decelerated Clinical Year (e.g., 3rd year over two years vs one):	[open-text response]
Extra Exam Time (e.g., Shelf Exams, OSCEs):	[open-text response]
Exam Reduced Distraction Environment:	[open-text response]
Intermediary or assistant to facilitate patient exam:	[open-text response]
Release from clinic to attend appointments:	[open-text response]
Release from Overnight Call:	[open-text response]
Scribe:	[open-text response]
Sign Language Interpreter or Cued Speech:	[open-text response]
Specialized Clinical Placement Site:	[open-text response]
Specialized Phone or Pager:	[open-text response]
Other (please enter total number and then enter details below):	[open-text response]

[a] *Note: Survey questions were originally designed by two experts in medical school disability administration (Drs. Neera R. Jain and Lisa M. Meeks) based on provisions of the Americans with Disabilities Act and disability research, and subsequently pilot tested by institutionally designated disability administrators from five medical schools to be refined for clarity.*

© 2023 Pereira-Lima K et al. *JAMA Network Open.*

eMethods 2. Number of medical schools meeting inclusion criteria in analyses for outcomes related to disability prevalence and accommodation use by study wave.

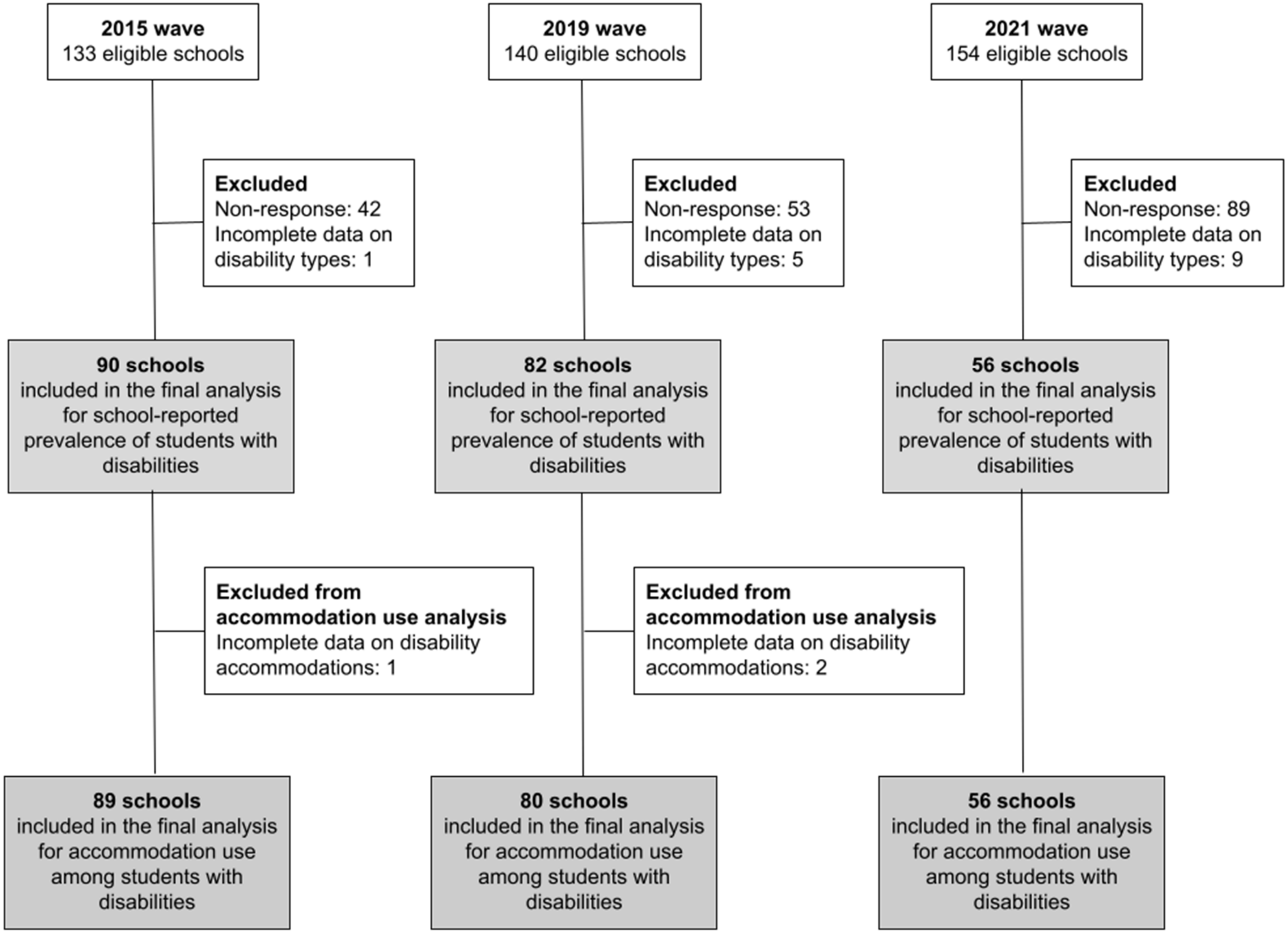

© 2023 Pereira-Lima K et al. *JAMA Network Open.*
