# Supplementary material for: Prevalence of Disability and Use of Accommodation Among US Allopathic Medical School Students Before and During the COVID-19 Pandemic
Source: JAMA Netw Open. 2023 Jun 14;6(6):e2318310. doi: 10.1001/jamanetworkopen.2023.18310 (PMC10267761; doi:10.1001/jamanetworkopen.2023.18310)
Supplement: Supplement 2. — Data Sharing Statement [file jamanetwopen-e2318310-s002.pdf]

# Data Sharing Statement

Pereira-Lima. Prevalence of Disability and Use of Accommodation Among US Allopathic Medical School Students Before and During the COVID-19 Pandemic. *JAMA Netw Open*. Published June 14, 2023. doi:10.1001/jamanetworkopen.2023.18310

**Data**
**Data available:** No

**Additional Information**
**Explanation for why data not available:** Our IRB does not allow for data sharing
